# The Neural Correlates of Embodied L2 Learning: Does Embodied L2 Verb Learning Affect Representation and Retention?

**DOI:** 10.1162/nol_a_00132

**Published:** 2024-06-03

**Authors:** Ana Zappa, Deidre Bolger, Jean-Marie Pergandi, Raphael Fargier, Daniel Mestre, Cheryl Frenck-Mestre

**Affiliations:** Institute of Neurosciences and Department of Cognition, Development and Educational Psychology at University of Barcelona, Barcelona, Spain; Cognition and Brain Plasticity Unit, Bellvitge Biomedical Research Institute, L’Hospitalet de Llobregat, Barcelona, Spain; Aix Marseille Univ, CNRS, LPL, Aix-en-Provence, France; Aix Marseille Univ, CNRS, ISM, Marseille, France; Université Côte d’Azur, CNRS, BCL, France

**Keywords:** EEG, embodied cognition, L2 learning, time frequency, virtual reality

## Abstract

We investigated how naturalistic actions in a highly immersive, multimodal, interactive 3D virtual reality (VR) environment may enhance word encoding by recording EEG in a pre/post-test learning paradigm. While behavior data have shown that coupling word encoding with gestures congruent with word meaning enhances learning, the neural underpinnings of this effect have yet to be elucidated. We coupled EEG recording with VR to examine whether embodied learning improves learning and creates linguistic representations that produce greater motor resonance. Participants learned action verbs in an L2 in two different conditions: specific action (observing and performing congruent actions on virtual objects) and pointing (observing actions and pointing to virtual objects). Pre- and post-training participants performed a match–mismatch task as we measured EEG (variation in the N400 response as a function of match between observed actions and auditory verbs) and a passive listening task while we measured motor activation (mu [8–13 Hz] and beta band [13–30 Hz] desynchronization during auditory verb processing) during verb processing. Contrary to our expectations, post-training results revealed neither semantic nor motor effects in either group when considered independently of learning success. Behavioral results showed a great deal of variability in learning success. When considering performance, low performance learners showed no semantic effect and high performance learners exhibited an N400 effect for mismatch versus match trials post-training, independent of the type of learning. Taken as a whole, our results suggest that embodied processes can play an important role in L2 learning.

## INTRODUCTION

Current theories of embodied cognition contend that language and motor processing are intertwined rather than independent, based on the assumption that cognition is grounded in multimodal representations originating in human experience (Barsalou, 2008; Pulvermüller, 2005). As concerns language, this entails that modal representations replace amodal symbolic linguistic representations (Fodor, 1983), giving motor processes an essential role in language processing (Wilson & Golonka, 2013). Indeed, sensory and motor systems are recruited during lexical processing, both during development (James & Swain, 2011) and in adults (Hauk et al., 2004; Pulvermüller, 1999, 2005; Thompson-Schill, 2003). Furthermore, neuroimaging has revealed an overlap in neural mechanisms for processing speech and hand movements (Nishitani et al., 2005). Along the same lines, gestural studies have suggested that gesture and speech comprise an integrated system (Goldin-Meadow, 2011; Graziano & Gullberg, 2018). Language processing is facilitated by gesture such that language-action congruency that occurs early in sentence processing can prime lexical retrieval (Glenberg & Kaschak, 2002). Furthermore, the retrieval of stored semantic representations directly influences sensorimotor activation as indexed by greater motor preparation when congruent action language is presented prior to movement (Aravena et al., 2012). Importantly, incongruity between actions and meaning can cause interference in meaning retrieval (Aravena et al., 2010; Barsalou, 1999). These results suggest that motor representations are not simply reactivated by linguistic representations postlexically (Mahon & Caramazza, 2009), but play an active part in meaning representation. In the current investigation, we examined the interaction between motor and semantic processes and how it may affect the mapping of novel action verbs to physical actions. Word encoding was coupled with compatible physical actions in an interactive virtual environment allowing for pseudonatural movements, to test whether motor activation enhances novel action word learning in a foreign language. We examined whether action verbs learned with specific actions produce greater motor activation post-training, as revealed by a decrease in the beta and mu band power, compared to verbs learned without accompanying actions. Finally, we examined whether our experimental manipulation led to improved retention as a result of a stronger motor trace in memory (Engelkamp & Krumnacker, 1980).

Encoding new words is an essential part of language learning and has been addressed in various learning studies that investigated cortical changes associated with learning, whether in the native language (L1) or in a second language (L2). In a seminal study, McLaughlin and colleagues (2004) found differences in L2 learners’ cortical activity after 14 hours of classroom instruction when processing newly learned L2 words, compared to pseudowords, as indexed by an N400 effect (McLaughlin et al., 2004). Importantly, the N400 modulation was found despite participants showing chance level accuracy behaviorally. However, N400 modulations due to semantic processing, as revealed in a primed lexical decision task, were only observed after 4 months of classroom instruction. In another study, the establishment of lexical representations for newly learned L2 words was revealed by an increase in N400 amplitude for L2 words after one semester of learning (Soskey et al., 2016). While these studies showed changes in the cortical response to newly learned L2 words, they investigated effects after extended L2 training. They did not allow for the observation of cortical changes occurring during the very first stages of encoding. In this vein, rapid cortical changes have been observed as a result of contextual word learning (Borovsky et al., 2010, 2012; Mestres-Missé et al., 2007; Shtyrov et al., 2010). Changes in N400 amplitude, suggesting meaning integration (Kutas & Federmeier, 2011), have been reported after as few as three exposures to novel words in highly constraining sentential contexts in the L1 (Mestres-Missé et al., 2007). Borovsky et al. (2010) concluded from their event-related potential (ERP) data that a single exposure to nouns in highly constraining contexts is sufficient to extract information concerning semantic restrictions. Subsequently, Borovsky et al. (2012) reported significant N400 modulations in a primed lexical decision task for both known and newly learned words. For newly learned words, the N400 effect was reported to be restricted to words learned in highly constrained contexts, as shown in independent pairwise comparisons. Finally, Bakker and colleagues (2015) found that ERPs only showed effects of lexicalization after a 24-hour period of consolidation (Bakker et al., 2015). From the above studies we can conclude that the neural response associated with semantic encoding can be modified following relatively little exposure. Nonetheless, the neural underpinnings of learning after a short training period have not yet been fully explored. The current study aims to help to fill this void by observing cortical activity after a 2-day, explicit, word-learning training using physical movement.

The benefit of physical movement for memorization and language learning is well established (Moskowitz, 1976; Quinn Allen, 1995). Outside of the language domain, a number of studies have shown that participants encode new information better when they perform gestures that are congruent with the new content. Physical activity—more than verbalization—facilitated the integration of sung melodies (Wakefield & James, 2011), as well as mathematical (Kontra et al., 2015) and scientific principles (Johnson-Glenberg et al., 2016; Johnson-Glenberg & Megowan-Romanowicz, 2017). Behavioral studies dating back to the 1980 s have shown that illustrative gestures support language retention better than other conditions (Engelkamp & Krumnacker, 1980; Engelkamp & Zimmer, 1984). For example, Engelkamp and Krumnacker (1980) showed that verb phrases such as “shuffle the cards” were better memorized when participants performed representative gestures during learning compared to either watching someone else perform the action, imagining the action or simply listening to the sentence. This *enactment effect*, has been replicated in L2 and artificial language word-learning studies. After 20–30 minutes of learning novel words by simply pointing to or touching the corresponding objects, participants showed associations between sensorimotor experiences (the location of an object in a vertical space) and the novel words (Öttl et al., 2017).

It has been argued that truly *embodied learning* involves self-performed or self-generated action, as opposed to simply observing or imagining action (James & Bose, 2011; James & Swain, 2011; Johnson-Glenberg, 2017, 2018; Johnson-Glenberg & Megowan-Romanowicz, 2017). In other words, highly embodied learning generally implies that learners physically perform gestures or movements that are directly linked to the content they are learning (Johnson-Glenberg, 2018). Both L1 and L2 lexical encoding studies generally use representative or iconic gestures (McNeill, 1992) that illustrate and map onto meaning directly. Studies with both adults (de Nooijer et al., 2013; Macedonia & Knösche, 2011) and children (Tellier, 2008) have shown that the production and recall of (L2) lexical items is enhanced by performing representative gestures.

The studies cited above indicate that action boosts memory performance and therefore supports language encoding. However, the cognitive processes that underlie this facilitation remain to be explained. One explanation is that physical action relays and helps establish implicit knowledge. Indeed, we often express information without realizing it through gestures (Church & Goldin-Meadow, 1986). According to Sun and colleagues (2001), what they describe as the “synergy” between explicit and implicit performance can aid in learning new skills (Sun et al., 2001, p. 1). The theory of Hebbian associative learning claims that the synchronous activity of neurons forms neuronal assemblies (Hebb, 1949); hence when lexical items are acquired along with action, cortical areas involved in language processing and those involved in action planning and execution quickly develop into shared neural circuits (Pulvermüller, 1999, 2005; Tomasello et al., 2018). To better understand how learning may be enhanced by movement, several studies have examined the neural underpinnings of lexical-motor interactions. In two functional magnetic resonance imagining (fMRI) studies, results showed that directly interacting with objects (James & Swain, 2011) and/or performing meaningful gestures (Macedonia et al., 2011) led to activation in the motor system during the subsequent auditory processing of newly learned lexical items. Moreover, performing iconic gestures during the learning of new lexical labels led to greater activation of the semantic network or “deeper semantic encoding” (Krönke et al., 2013, p. 1).

Despite the importance of the above studies, fMRI may not be the ideal tool to show motor-to-language effects or vice versa. Indeed, much debate surrounds the role of motor activation during language processing. One of the arguments against embodied semantics is that language-induced motor activations are postlexical and not a necessary part of language processing (Mahon & Caramazza, 2008). High temporal resolution—an advantage of electroencephalography (EEG) compared to fMRI—is hence an important element when arguing for embodied language representations. One way of quantifying motor cortex activity is to use EEG to measure event-related synchronization/desynchronization (ERS/ERD) via stimulus-locked time-frequency analysis (Vukovic & Shtyrov, 2014). A decrease in alpha, mu (8–13 Hz), and beta-band (13–30 Hz) power, mostly over central or centroparietal sites, has been associated with sensorimotor activation involved in movement preparation and execution (Niccolai et al., 2014; Pfurtscheller & Lopes da Silva, 1999; Pineda, 2005). A decrease in the alpha rhythm has likewise been linked to motor imagery (Höller et al., 2013). Recently, a decrease in energy in frequencies associated with motor processes has also been observed during action language comprehension. Reading sentences describing manual actions versus abstract sentences led to the suppression of mu rhythms at frontocentral sites (Alemanno et al., 2012; Moreno et al., 2015). This does not entail a one-to-one mapping between power decreases in specific frequency bands and specific cognitive functions. However, it does reveal an association between mu and beta oscillations over central and centroparietal sites and motor/sensorimotor activity, which can be used to index language-motor interactions (cf. Klepp et al., 2019). To our knowledge, the only study that has used time frequency to measure motor activation during language processing pre- and post-training was conducted by Fargier and colleagues (2012). They showed that learning novel words in association with specific self-performed actions led to greater mu desynchronization over centroparietal sites post-training, which they interpreted as motor activation, compared to learning in association with abstract animations (Fargier et al., 2012). However, on the second day of training, a frontocentral distribution of the effect, as opposed to a typical centroparietal mu distribution, led the authors to conclude that it was confined to a convergence zone.

The above-described evidence points to the need to develop this line of investigation. Embodied cognition binds social and physical contexts to cognition, suggesting that the environment in which learning takes place could potentially play an important role in learning outcome (Black et al., 2012). According to Atkinson (2010), learning is not just a mental process but one that occurs in environments made up of “bodies, cognitive tools, social practices and environmental features” (p. 609) and this multimodality calls for an experimental approach that is likewise multimodal. One caveat of experimental protocols that examine embodied learning is that, given the need for control, movement is generally reduced to minimal hand actions and training most often occurs in isolated and decontextualized environments (Peeters, 2019). This is especially true of studies that analyze the neural correlates of language processing and learning using techniques such as fMRI, EEG, or magnetoencephalography (MEG). For instance, when interaction with objects has been made possible, it has been limited to pointing at or touching objects, hence making it impossible for participants to map specific actions to specific words. Given the importance of interlocutors, social context, and physical cues on how language is understood in real life (Knoeferle, 2015), physical and environmental limitations likely affect how language is learned. Within the framework of embodied cognition, it is especially important to take a closer look at the gap between real-life language processing and that which takes place in an experimental environment (Tromp et al., 2018).

Virtual reality (VR) is an important tool for investigating embodied language learning. In a 3D environment, participants are presented with auditory and visual stimuli, including objects with which they can interact while receiving real-time feedback for their actions via a graphic rendering system (Zappa et al., 2019). Numerous L2 studies have used virtual environments and VR paradigms involving varying degrees of immersion to investigate language learning. They have generally found facilitation for learning in immersed conditions compared to word–word or picture–word paired associations (Berns et al., 2013; Lan et al., 2015). Furthermore, participants who learned in a virtual environment using avatars (*Second Life*; Linden Labs, 2024) showed neural activations that were more distributed and associated with a more embodied brain experience compared to the control group (Lan et al., 2015). However, *Second Life* paradigms are limited when it comes to exploring truly interactive embodied learning (for a review of L2 video games, see Legault et al., 2019). To overcome this, Legault and colleagues (2019) taught participants a set of L2 words in an ecologically valid immersive VR zoo or kitchen, using word–word paired association as a control. In the immersive VR condition, they wore a head mounted display and encountered new words within their contexts (i.e., kitchen items in a kitchen). Participants—especially less successful learners—showed higher accuracy in the immersive VR condition (Legault et al., 2019).

Peeters (2019) claimed that VR “shifts the theoretical focus toward the interplay between different modalities … in dynamic and communicative environments, complementing studies that focus on one modality in isolation.” (p. 1) VR uses visual and auditory stimuli to create an immersive sensory experience, providing participants with credible environments. In addition, participants’ head and body movements are tracked by input tools (e.g., hand controls) and participants are given real-time feedback for their actions, which provides a sensation analogous to real life (Burdea & Coiffet, 2003). The fact that participants can interact with the environment by manipulating virtual objects and carrying out naturalistic actions gives them a sense of agency (Johnson-Glenberg, 2018). Compared to traditional experiments, this leads to a greater implication of the sensorimotor system, with responses and actions being closer to what occurs in real life (Bohil et al., 2011). Finally, VR combines ecological validity with full control over the onset, location, and duration of presentation of the multimodal stimuli. Very few studies have paired virtual reality with EEG to study language processing. In an exploratory EEG-VR experiment, participants listened to a sentence (“I just ordered this salmon”) and saw a virtual object that either matched (salmon) or mismatched (pasta) the object in the sentence. An N400 effect was observed for mismatched versus matched pairs, and the authors interpreted this as proof of validity for combining VR and EEG to examine language processing (Tromp et al., 2018). However, participants did not manipulate objects and the involvement of the motor cortex was not examined. Recently, Zappa and colleagues measured motor-related EEG activity in an interactive VR environment while participants performed a go/no-go task and listened to action verbs prior to executing the corresponding actions. Motor activation was found via a decrease in power in the mu and beta bands during verb processing and prior to movement proper, providing compelling evidence in a naturalistic setting of how motor and linguistic processes interact (Zappa et al., 2019). Moreover, greater ERD was found for Go trials, suggesting that motor preparation influenced semantic processing. These results provide the basis for the present study, investigating the association of new linguistic labels to motor actions.

Our study used a combined EEG-VR methodology to explore the neural correlates of embodied learning. EEG and VR were not employed simultaneously, but EEG was used to measure learning pre- and post-training and VR was used to facilitate embodied and situated learning. Using a head mounted VR system (Oculus Rift) and controller, participants were exposed to an auditory L2 lexicon of action verbs associated with videos of congruent physical actions. They were assigned to one of two groups, according to whether they were required to perform a specific motor action that corresponded to the observed action, the specific action condition (e.g., pick up an object and throw it for the verb “throw”) or the pointing condition (point to the object). Both pre- and post-training, learners’ knowledge of the semantic meaning of the training verbs was measured behaviorally and through EEG using a match–mismatch task. Motor resonance was also measured using EEG while participants listened to the training verbs as well as a set of filler verbs that were never taught, both pre- and post-training (See Table 1). We expected motor resonance during auditory verb processing to vary as a function of learning condition during the post-training session but not the pre-training session. We hypothesized that verbs learned with specific actions would be encoded with a stronger motor trace and hence produce greater motor activation than verbs learned in the pointing condition. We also predicted that embodied learning using specific self-performed congruent physical actions would lead to better learning outcomes post-training compared to the pointing condition, as revealed by behavioral accuracy.

**Table 1.** Stimuli

**Serbian word**	**English translation**	**French translation**
gurni	push	pousser
zagrebi	scratch	gratter
pusti	drop	lacher
baci	throw	lancer
okreni	pivot	faire pivoter
premesti	move	déplacer
protresi	shake	secouer
kucni	tap	tapoter
uhvati	catch	attraper
podigni	lift	soulever
lupi	hit	cogner
obori	tip over	faire tomber

### Hypotheses

1. In accordance with the theory that learning lexical items along with action can form shared neural networks (Pulvermüller, 1999, 2005; Tomasello et al., 2018) and studies showing greater motor activation for object labels learned with direct object interactions (James & Swain, 2011) or specific self-performed actions (Fargier et al., 2012), we expected to find a decrease in beta (13–30 Hz) and mu (8–13 Hz) band power (motor activation) post-training compared to pre-training during the processing of the training verbs (passive listening task). Given that only training verbs were associated with meaning, these effects were not expected to be observed for filler verbs, for which no variation pre–post training should have occurred.
2. Activity in the premotor context has been found when learners process verbs learned with iconic gestures but not those learned with meaningless gestures (Macedonia et al., 2011). We therefore expected to find greater motor resonance for verbs learned in the specific action group compared to the pointing group.
3. Studies have shown that learners associate a new word-form to semantic content after very little exposure (Borovsky et al., 2012; Mestres-Missé et al., 2007; Yum et al., 2014). During the match–mismatch task, we expected that pre-training, we would not find an N400 effect for match versus mismatch trials. Post-training, we expected to find greater N400 amplitude for mismatch versus match trials in both learning groups, due to participants accessing the semantic meaning of newly learned verbs.
4. Along with studies in nonlinguistic domains showing enhanced learning when gestures are used (Broaders et al., 2007; Johnson-Glenberg et al., 2016; Johnson-Glenberg & Megowan-Romanowicz, 2017; Kontra et al., 2015; Wakefield & James, 2011), both behavioral and electrophysiological evidence from L2 learning studies has revealed that congruent gestures support linguistic memory and encoding and improve performance (de Nooijer et al., 2013; Macedonia et al., 2011; Macedonia & Knösche, 2011; Mayer et al., 2015; Tellier, 2008). We therefore hypothesized that the N400 effect outlined in Hypothesis 3 would be greater for the specific action group compared to the pointing group.
5. In accordance with Hypotheses 3 and 4, we expected to find a positive correlation between greater motor resonance during the passive listening task and a greater N400 amplitude for mismatch versus match trials in the match–mismatch task.
6. In accordance with Hypothesis 4, we predicted that our behavioral results would show greater accuracy for verbs learned in the specific action condition compared to the pointing condition.

## MATERIALS AND METHODS

In the current study we manipulated the type of action performed (specific object manipulation vs. pointing) during L2 learning in a VR environment. During learning, participants saw movements performed by a virtual hand. The specific action group reproduced the movement on a virtual object and the pointing group pointed to the virtual object on which the action was performed. EEG was recorded both pre- and post-training.

### Ethics

This research complies with all relevant ethical regulations and has been approved by the local university ethics committee.

### Statistical Power Analysis

For Hypothesis 1, a statistical power analysis was performed for sample size estimation using G*Power 3.1 (Faul et al., 2009). The analysis was based on data from a previous published study (Zappa et al., 2019; *N* = 20), comparing decrease in mu and beta band power for no-go vs. go trials. The effect size in this study was 0.8, which is considered to be large using Cohen’s (1988) criteria. However, given that large effect sizes are often overestimated and rare, we selected a medium effect size (0.5) for our power analysis using a Cohen’s *d*. With an alpha = 0.05 and power = 0.80, the projected sample size needed is approximately *N* = 34 for the simplest within group comparison (Hypothesis 1). Thus, our proposed sample size of 42 is adequate for the three within-subjects comparisons (Hypotheses 1, 3, and 5) as they concern a decrease in power in either the mu/beta bands or the N400 effect, which has been shown to require a smaller sample than 42 where semantic effects are concerned (Borovsky et al., 2012; Fields & Kuperberg, 2020; Tromp et al., 2018). Note, however, that this is not the case for more subtle N400 effects, such as those resulting from pre-activating the phonological form of upcoming words (Nicenboim et al., 2020) or determiner gender (Nieuwland et al., 2018). As regards Hypotheses 2, 4, and 6, we ran a power analysis using Cohen’s *d* that calculated the estimated power for our between-subjects analysis, given a sample size of 42 per condition and a medium effect size (0.5). The estimated power came out to 0.60. The final sample size of 42 as concerns the between-participant hypotheses is therefore motivated by practicality (budgetary and time constraints) as well providing sufficient power for the within-subjects hypothesis.

### Participants

Eighty-four (42 per group) right-handed French native speakers (aged 19–26) with no previous knowledge of Serbian or related languages participated in the study. Four participants’ data were excluded due to excessively noisy data, leaving 39 participants in the specific action group and 41 in the pointing group. Participants were volunteers from the student population of the Aix-Marseille University, having no history of neurological insult. All participants gave their written informed consent prior to the experiment and received 40 euros for their participation. Data collection was inadvertently commenced prior to in principle acceptance, before the analysis plans had been finalized.

### Stimuli

**Figure 1.**
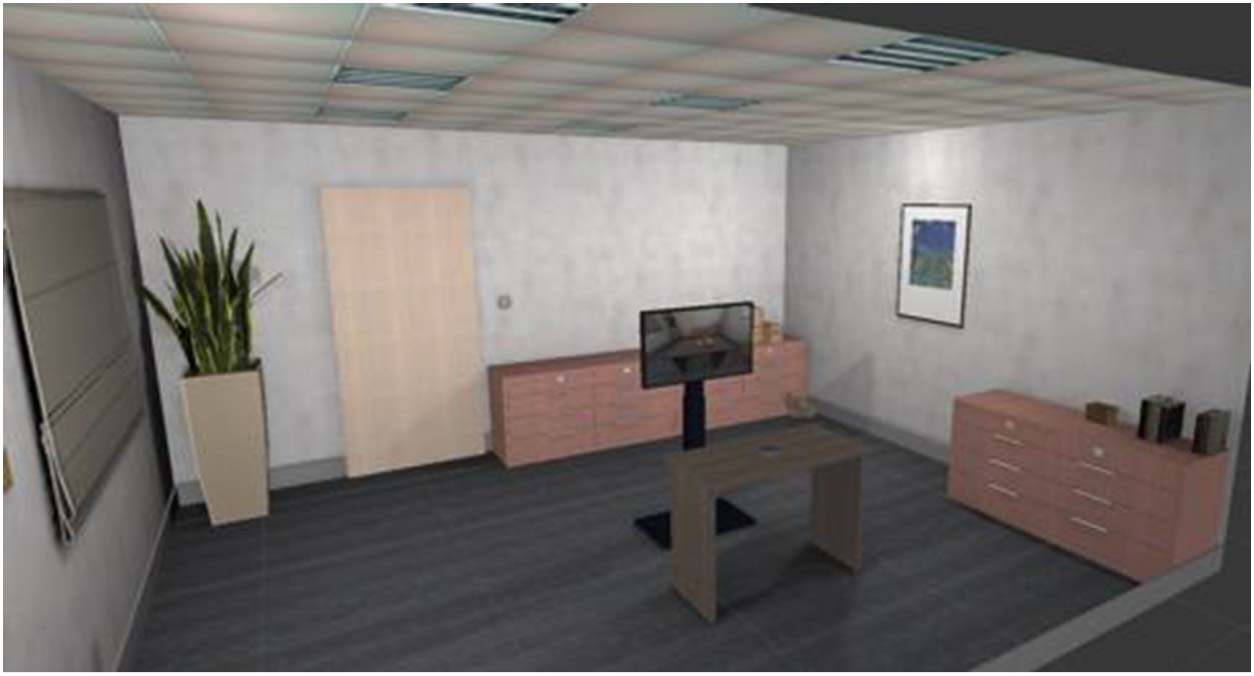
Virtual reality environment and screen.

Auditory stimuli consisted of 12 imperative transitive verbs in Serbian that were not transparent with their translation equivalents in French, Spanish, Italian, Portuguese, German, or English. Serbian is a South Slavic language that is linguistically distant from both Romance and Germanic languages such that transparency posed little threat. The verbs denoted actions that can be performed using one’s hand and arm, and were previously validated in a VR environment (Zappa et al., 2019): /ˈgurni/ [push], /zαˈɡrεbiː/ [scratch], /ˈpuːstiː/ [drop], /ˈbαtsiː/ [throw], /oˈkrεniː/ [pivot], /ˈprεmεstiː/ [move], /ˈkuːtsniː/ [tap], /ˈuxυαtiː/ [catch], / podiɡniː/ [lift], /ˈluːpiː/ [hit], /oˈboriː/ [tip over], /proˈtrεsiː/ [shake]. Verbs were recorded in a professional sound booth by two native female speakers of Serbian. Learners learned with words recorded by one of the speakers and were tested post-training with words recorded by the second speaker, to avoid familiarity effects. Participants in both groups (specific action vs. pointing) heard Speaker 1 during training and Speaker 2 during EEG testing. A set of 12 filler verbs denoting different actions was recorded for the passive listening EEG task. Visual stimuli for learning consisted of an office environment containing a 3D 10-point star polygon and a CRT screen (Figure 1).

Animations of hand and arm movements corresponding to the training verbs, performed on the 3D 10-point star polygon were recorded. These animations were used in both learning conditions to teach participants the movements that corresponded to the verbs and for the match–mismatch task pre- and post-training.

### Learning Apparatus

An Oculus VR headset and controller was used for training purposes. The Oculus headset visually immerses participants by presenting them with a 360-degree visual scene and 3D virtual objects. The controller allowed participants to manipulate objects while motion capture was recorded online.

### Software

During pre- and post-tests, StimPres (Tufts University) was used for stimulus presentation on a desktop computer and a 64-channel Biosemi system (Actiview) was used for acquisition. Unity (Unity Technologies, 2024) software controlled virtual object presentation during learning.

### General Procedure

The experiment took place over 2 days. On the first day, participants underwent EEG and behavioral pre-tests followed by a VR learning session. On the second day, participants took part in a second VR learning session with the same materials as Day 1, followed by the EEG recording and behavioral post-tests. Learning sessions lasted roughly half an hour each day.

#### Learning procedure

**Figure 2.**
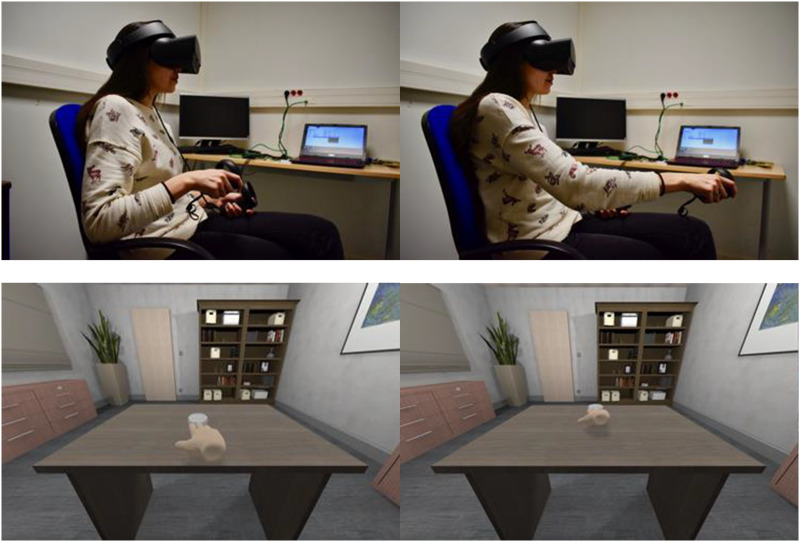
Top: A participant wears an Oculus headset and performs the verb “gurni” [push]. Bottom: The participant’s movements translate into the virtual hand pushing the virtual object away. Photo courtesy of Ana Zappa.

Participants were comfortably seated at a desk wearing a VR Oculus headset and holding a controller. Participants in both the specific action and the pointing groups were presented with an auditory verb and requested to overtly repeat the verb prior to observing an action on the virtual CRT screen within the VR environment. Following this, a virtual object appeared on the virtual desk. The specific action group manipulated the object via the controller, performing the action observed on the virtual screen (Figure 2). The pointing group pointed to the object. Participants were exposed to each verb six times during the first session (Day 1) and four times during the second session (Day 2). The number of exposures was established after pilot studies showed that some participants showed at ceiling accuracy post-training after eight and six exposures.

#### EEG procedure

EEG was recorded during both pre- and post-tests. Participants were comfortably seated at a desk situated 60 cm away from a computer screen in an electrically shielded sound-attenuated booth.

##### Passive listening task.

During the first task participants were asked to listen to the list of verbs passively, with no associated task. They heard the 12 verbs used for learning and 12 filler verbs, three times. A trial began with an ocular fixation cross displayed in the center of the computer monitor for 200 ms prior to and for the duration of the auditory word, which was presented via electrically shielded speakers. A visual “blink” prompt was displayed immediately thereafter for 2 s. The experimental session lasted roughly 10 min.

##### Match–Mismatch task.

During the match–mismatch task the auditory verbs used in learning were preceded by either the compatible (match) or an incompatible (mismatch) animation. A question mark appeared directly following the auditory verb. Participants were required to answer yes or no on a response box. A visual blink prompt was displayed immediately thereafter for 2 s. The experimental session lasted roughly 25 min, including one break.

#### Behavioral procedure

Binary behavioral responses (match/mismatch) and response times were recorded during the match–mismatch task. In an exploratory manner, word retention was tested behaviorally in two tasks, after each training session.

In the translation task, participants listened to the learned verbs and were asked to provide a French translation for each one. For the production task, participants saw the animations of the verbs they learned and were asked to write the learned words phonetically.

### EEG Data Acquisition

During pre- and post-tests, EEG activity was recorded continuously from 64 scalp electrodes located at midline as well as left and right hemisphere positions over frontal, central, parietal, occipital, and temporal areas by means of a 64-channel electrode cap mounted with silver-chloride active electrodes (BioSemi Active Two system AD box). During acquisition, the offset of the electrodes was maintained within the ±20 mV range, in line with common practice using active electrode setups, and data were sampled online at 512 Hz. Blinks and vertical eye movements were monitored via an electrode placed under the right eye and horizontal eye movements were monitored via an electrode placed at the outer canthus of the left eye. One electrode was placed over each mastoid. EEG was recorded continuously during the experiment and periods spanning from −150 pre-stimulus onset to 1,100 ms post-stimulus onset were used post-recording for analyses.

### EEG Data Processing

EEG data were bandpass filtered between 0.1 and 40 Hz using a 1,408-order finite impulse response filter windowed (Kaiser) sinc filter. The filtered data were re-referenced offline to the average of the two mastoids. To detect noisy electrodes, we applied several approaches to take into account different noise sources, such as muscle artifacts, electrode pops, ocular movements, and so forth. First, based on the continuous data, we identified those electrodes whose amplitudes exceeded a pre-defined threshold of ±50 mV and, for each electrode, the total above-threshold time was calculated. In addition, we determined those electrodes with extreme amplitudes by calculating the robust *z*-score, as described by Bigdely-Shamlo and colleagues (Bigdely-Shamlo et al., 2015). The robust *z*-score is calculated based on the median and the robust standard deviation (*z*_robust_ = 0.17413 * interquartile range) and those electrodes with a *z*_robust_ > 5 were marked as bad. We also tested the electrodes based on the noisiness criterion described by Bigdely-Shamlo and colleagues (Bigdely-Shamlo et al., 2015), which calculates the ratio of the power of high frequency signal components to the power of low frequency components. This was complemented by visual examination of the power spectral density of each electrode to determine those with excessive low and high frequency activity or contaminated by line noise. We performed a baseline correction using a 150 ms pre-stimulus period for both the time frequency and ERP analyses.

Noisy electrodes marked for rejection were removed. To correct for ocular movements, sections of the EEG signal that were highly contaminated with noise were removed from the dataset before carrying out independent components analysis (ICA). ICA was carried out on the continuous data of each participant. Principal component analysis (PCA) was applied prior to ICA computation to reduce the dimension of the data and accelerate the ICA computation time. The number of PCA components was estimated by calculating the explained variance of each principal component and conserving only those principal components explaining 99% of the variance. Those independent components corresponding to eye blinks were identified automatically and rejected. Once the ocular artifacts were corrected using ICA, the rejected electrodes were interpolated using spherical spline interpolation. The data were then segmented and epochs were visually inspected. Those contaminated by noise were removed. The epoched data were then divided into separate conditions for analysis.

### EEG Data Analysis

#### ERPs

The ERP data were modeled using linear mixed effect models for the mean voltage amplitudes in the established N400 window, between 300–600 ms (Kutas & Federmeier, 2011; Tromp et al., 2018), time locked to the onset of the verb. Analyses for the N400 component were conducted on the data acquired at 35 electrodes, including five over midline (Fz, FCz Cz, CPz, Pz), and 30 lateral electrodes divided equally over the left (F1, F3, F5, FC1, FC3, FC5, C1, C3, C5, CP1, CP3, CP5, P1, P3, P5) and right (F2, F4, F6, FC2, FC4, FC6, C2, C4, C6, CP2, CP4, CP6, P2, P4, P6) hemispheres. A linear mixed effects regression model (lmer) including the fixed factors Group (Specific Action vs. Pointing), Session (Pre vs. Post), Condition (Match vs. Mismatch), and Region of Interest (ROI; Midline and Left and Right lateral electrodes), as well as their interactions, was performed. Participant and Item both included random intercepts. Condition included a random slope for Participant and, if model converged, for Item. The fixed factors were sum coded to allow for the interpretation of main effects.

In an exploratory analysis to determine where significant differences between match and mismatch conditions emerged, a permutation test with false discovery rate correction was carried out on all time points of the post stimulus interval for each electrode. A significant difference was only considered (*q* ≤ 0.05) if its duration exceeded 10 ms (∼5 consecutive time samples for a sampling frequency of 512 Hz).

#### Event-related spectral perturbation

To test the hypothesis of a difference in event-related spectral perturbation (ERSP) between filler and test verbs pre- vs. post-training the data were modeled using linear mixed effect regressions, with the LmerTest package (Kuznetsova et al., 2017) implemented in R for activity in the mu, high beta, and low beta bands. The event-related spectral perturbation (ERSP) was calculated on the data from the passive listening task, time-locked to the onset of the verb, using the MNE-Python software (Gramfort et al., 2013).

Based on our previous study (Zappa et al., 2019), analyses included nine frontocentral electrodes associated with motor processes (FC3, FC4, C3, C4, CP3, CP4, FCz, Cz, and CPz). Before computing the ERSP, individual trials for each participant were padded. This was carried out to deal with the short baseline duration (150 ms), which made it difficult to resolve the lower frequencies of interest, and to limit edge effects. Padding was carried out by concatenating 1 s of data composed of repetitions of the first data sample of the baseline interval to the start of each trial. This padding yielded a baseline interval with a duration of 1,150 ms.

To compute the ERSP, time frequency decomposition was effectuated at the single trial level for each participant and each condition (pre-training, post-training, test verbs, filler verbs) by applying complex Morlet wavelets over the 5 Hz to 32 Hz frequency band. The number of wavelet cycles was varied linearly from 5 to 10 cycles as a function of frequency and yielded a FWHM (full width at half maximum) of 225 ms and 187 ms at 10 Hz and 12 Hz, respectively, (mu band), 175 ms at 15 Hz (beta band), and 135 ms at 25 Hz (upper beta band). A mean baseline was computed by averaging the trial-level baselines, this yielded a single mean baseline for both verb types (test verbs and filler verbs) and for each participant. The post-stimulus intervals of individual trials were expressed in terms of decibel change relative to the mean baseline (−150–0 ms).

An lmer model including the fixed factors Session (Pre vs. Post), Group (Specific Action vs. Pointing), Verb (Learned vs. Filler), and ROI (Midline: FCz, Cz, CPz; Left: FC3, C3, CP3; Right: FC4, C4, CP4), as well as their interactions, was performed. Participant and Item both included random intercepts. Condition included a random slope for Participant and for Item, provided the model converged. The fixed factors were sum coded to allow for the interpretation of main effects.

### Behavioral Data Analysis

A generalized lmer model (glmer) was used to examine accuracy in the match–mismatch task. The sum-coded fixed effects factors included Condition (Match vs. Mismatch), Session (Pre vs. Post-training), Group (Specific Action vs. Pointing), as well as their interactions. Participant and Item both included random intercepts, provided the model converged.

For the translation task, as participants never heard the French translations for the learned words, translations that described an action that matched the learned action were scored correct, independent of the actual verb used (i.e., “toss” or “throw”). For the production task, as participants were never shown the words in writing, words that were considered recognizable were scored as correct (i.e., “baci” or “batsi”). Words that began with the correct phoneme but were not recognizable as the Serbian word received a 0.5 score (i.e., “bafi”). Given that all words ended in the same phoneme [i], this did not apply to word terminations.

## RESULTS

### Declared Analyses

To determine the pattern of data, we ran a series of comparisons for both behavioral and electrophysiological measures using mixed effect models. We compared the data across sessions (pre- vs. post-training). When interactions emerged, we ran subsequent independent models (in each session, and for each group).

#### Match–mismatch task: ERP data

To test the hypothesis of a significant difference in N400 effect for match versus mismatch trials, post versus pre-training, we compared mean voltage amplitudes in the established N400 window, between 300–600 ms, time locked to the onset of the verb. An interaction between Condition, Group, and Session emerged. To test the hypothesis of an N400 effect for match versus mismatch post- (but not pre-) training, we modeled the data independently for pre-training and post-training. No effect of Condition was found for either group.

##### Pre- versus post-training.

We ran a first model which included the sum-coded fixed factors Session (Pre vs. Post), Group (Specific Action vs. Pointing), Condition (Match vs. Mismatch), ROI (Midline, Left, Right), as well as their interactions, with random intercepts for Participant and Item. Condition included a random slope for Participant and for Item, but as this model did not converge we applied the model without these slopes. The model revealed an interaction of Condition:Group:Session (*β* = 1.12, *SE* = 2.18, *t* = 5.145, *p* < 0.001). The data were modeled independently thereafter for each training session.

##### Pre-training.

The model included the sum-coded fixed factors Group (Specific Action vs. Pointing), Condition (Match vs. Mismatch), ROI (Midline, Left, Right), as well as their interactions, with random intercepts for Participant and Item. Condition included a random slope for Participant and for Item, but as this model did not converge we applied the model without these slopes. No effect of Condition was found (*β* = 1.42, *SE* = 2.54 *t* = 0.559, ns), nor was there an effect of Group (*β* = 2.39, *SE* = 3.25, *t* = 0.736, ns) or an interaction between the two (*β* = 1.79, *SE* = 1.57, *t* = 1.140, ns).

##### Post-training.

The model included the sum-coded fixed factors Group (Specific action vs. Pointing), Condition (Match vs. Mismatch), ROI (midline, left hemisphere, right hemisphere) and their interactions, with random intercepts for Participant and Item. Condition included a random slope for Participant and for Item but as this model did not converge, we applied the model without these slopes. No effect of Condition was found (*β* = 2.60, *SE* = 3.18 *t* = 0.89, ns), nor was there an effect of Group (*β* = 1.85, *SE* = 4.16 *t* = 0.443, ns) or an interaction (*β* = 1.90, *SE* = 1.95 *t* = 0.974, ns).

#### Passive listening task: Time-frequency data (mixed models)

Based on our hypothesis of greater desynchronization in the mu, low beta, and high beta bands post-training compared to pre-training for learned (but not filler) verbs and for both test and control groups, we carried out a post- versus pre-training comparison of the ERSP of filler and learned verbs. We tested the hypothesis of a difference in ERSP between filler and learned verbs post- versus pre-training, specifically greater desynchronization in the mu, high beta, and low beta bands post-training, compared to pre-training, for learned (but not filler) verbs, for both groups and possibly greater for the test group.

In a pre- versus post-training comparison, an interaction between Verb, Group, and Session emerged. We modeled the data independently for pre-training and no effect of Verb was found for either group. Post-training, an interaction between Verb and Group emerged. We modeled the data independently for both groups, and no effect of Verb was found for either group.

##### Pre- vs. Post-training.

The model included the sum-coded fixed factors Session (Pre vs. Post), Group (Specific Action vs. Pointing), Verb (Filler vs. Learned), as well as their interactions, and ROI (Midline, Left, Right), with random intercepts for Participant and Item. Verb included a random slope for Participant and for Item, but as this model did not converge we applied the model without these slopes. The model revealed a three-way interaction of Verb:Group:Session (*β* = −6.25, *SE* = 2.08, *t* = −3.00, *p* < 0.001). The data were modeled independently thereafter for each training session.

##### Pre-training.

The model included the sum-coded fixed factors Group (Specific Action vs. Pointing), Verb (Filler vs. Learned), ROI (Midline, Left, Right), as well as their interactions, with random intercepts for Participant and Item. Verb included a random slope for Participant and for Item, but as this model did not converge we applied the model without these slopes. No effect of Verb was found (*β* = −5.24, *SE* = 1.85, *t* = −0.283, ns).

##### Post-training.

The model included the sum-coded fixed factors Group (Specific Action vs. Pointing), Verb (Filler vs. Learned), ROI (Midline, Left, Right), as well as their interactions, with random intercepts for Participant and Item. Verb included a random slope for Participant and for Item. The model revealed an interaction of Verb:Group (*β* = 3.19, *SE* = 1.56, *t* = 2.045, *p* < 0.001). The data were modeled independently thereafter for each experimental group.

- **Specific Action group**: The model included the sum-coded fixed factors Verb (Filler vs. Learned) and ROI (Midline, Left, Right), with random intercepts for Participant and Item. No effect of Verb was found (*β* = −7.85, *SE* = 6.09, *t* = −0.129, ns).
- **Pointing group**: The model included the sum-coded fixed factors Verb (Filler vs. Learned) and ROI (Midline, Left, Right), with random intercepts for Participant and Item. No effect of Verb was found (*β* = −3.72, *SE* = 4.93, *t* = −0.756, ns).

#### Behavioral performance for match–mismatch task

**Figure 3.**
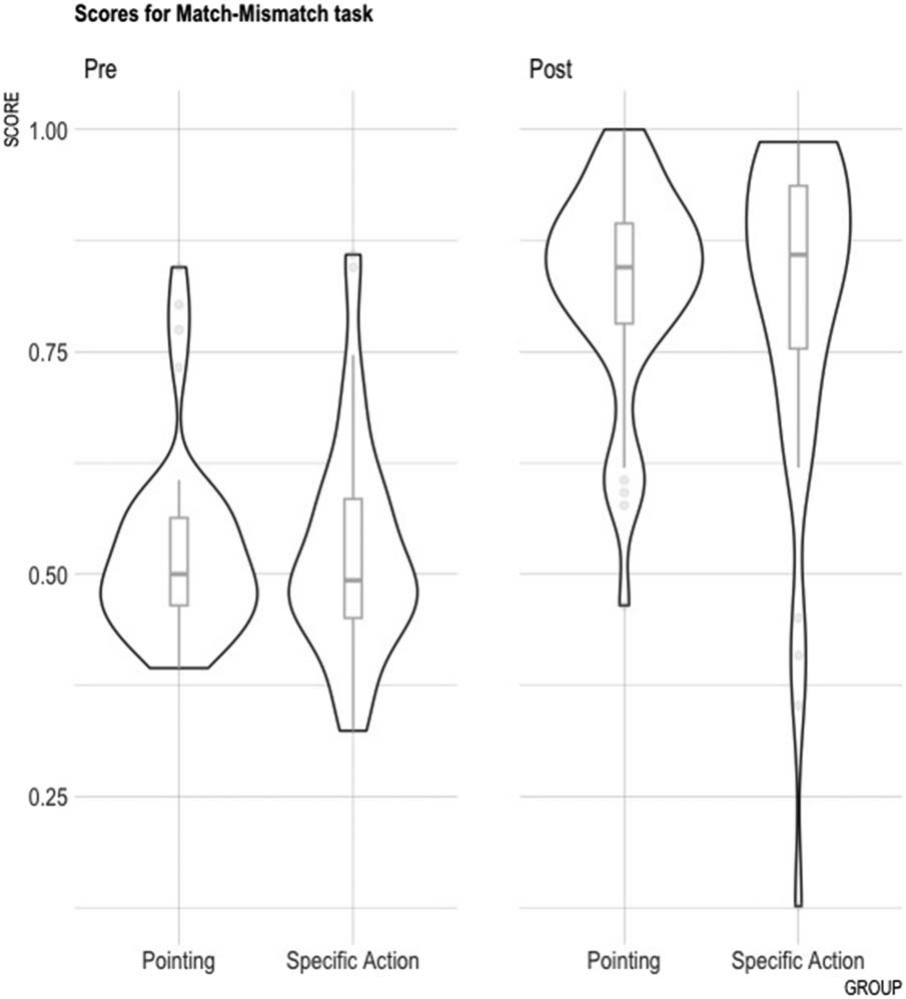
Match–mismatch scores pre- and post-training for Pointing and Specific Action groups.

To test the hypothesis of better behavioral accuracy post- versus pre-training and, in the post-training session, for the Specific Action versus Pointing groups, we compared behavioral accuracy during the match–mismatch task using a glmer model. A first model, comparing accuracy during pre- versus post-training sessions revealed an interaction of Condition by Session. Participants performed at chance pre-training and above chance post-training (Figure 3). When the post-training data were modeled separately, a significant effect of Condition emerged, but no effect of Group or interaction was found.

The first model included the sum-coded fixed factors Session (Pre vs. Post), Group (Specific Action vs. Pointing), Condition (Match vs. Mismatch), as well as their interactions, with random intercepts for Participant and Item. The model revealed an interaction of Condition:Session (*β* = −0.96, *SE* = 0.02, *z* = −4.174, *p* < 0.001). The data were modeled independently thereafter for each training session.

##### Pre-training.

The model included the sum-coded fixed factors Group (Specific Action vs. Pointing), Condition (Match vs. Mismatch), as well as their interaction, with random intercepts for Participant and Item. No effect was found of either Condition (*β* = 0.15, *SE* = 0.12, *z* = 0.559, ns) or Group (*β* = 0.824, *SE* = 0.066, *z* = 12.458, ns), nor of their interaction (*β* = −0.024, *SE* = 0.05, *z* = −0.439, ns).

##### Post-training.

The same model conducted on the post-training data revealed an effect of Condition (*β* = −0.44, *SE* = 0.10, *z* = −4.09, *p* < 0.001). The effect of Group was not significant (*β* = −0.04, *SE* = 0.139, *z* = 1.626, ns), nor was that of their interaction (*β* = 0.089, *SE* = 0.054 *z* = 1.626, ns).

### Exploratory Analyses

#### Behavioral performance, production scores, and translation

To measure learning, participants performed a written translation task (from Serbian into French) and a written production task (of the Serbian verbs) post-training.

**Figure 4.**
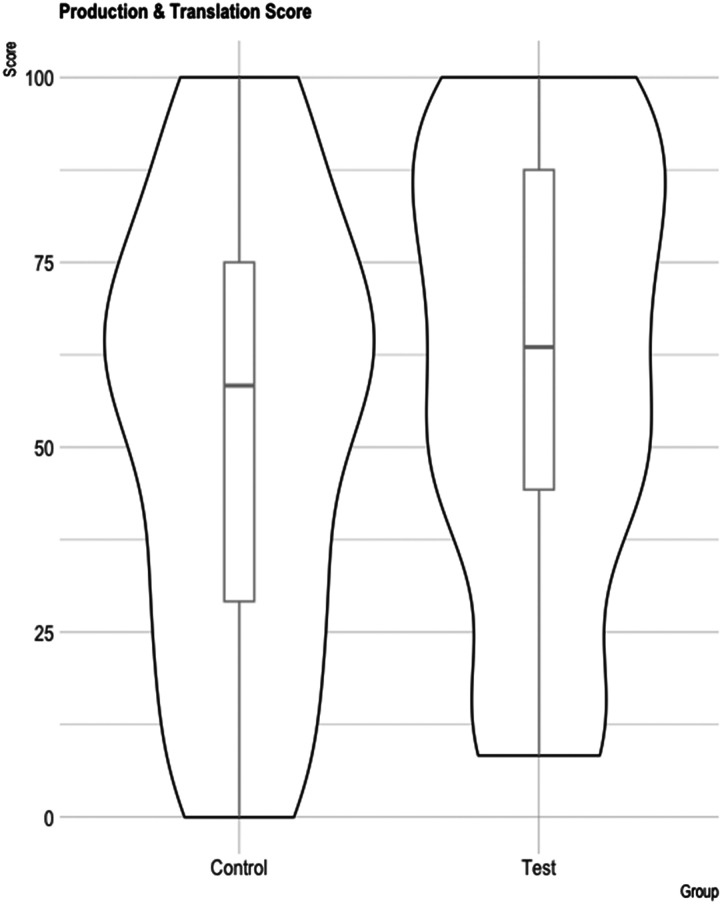
Production and translation test percentage post-training for Pointing and Specific Action groups.

As can be seen in Figure 4, both groups showed a great deal of variability in performance post-training. Given this variability, we separated both original groups (Specific Action and Pointing) into two Performance groups (High and Low performance learners). High performance learners scored above 50% accuracy for the average of the production and translation tasks; this subgroup included 55 learners (30 from the Specific Action group and 25 from the Pointing Group). Low performance learners scored below 50% accuracy and included 25 learners (9 from the Specific Action group and 16 from the Pointing group).

We then performed exploratory analyses on these two subgroups separately, which we report in the next section.

#### Match–mismatch task: High vs. Low performance learners

To test the hypothesis of a significant N400 effect for match versus mismatch trials post-training for the high performance (but not low performance) learners, we compared mean voltage amplitudes in the N400 window post-training across both groups. An interaction between Condition and Performance emerged. The data were modeled separately for each Performance group, revealing no effect of Condition for the low performance learners and a significant effect of Condition (N400 effect for mismatch vs. match trials) for the high performance learners. An interaction of Condition by Performance emerged, and we performed analyses on high performance participants across the two learning groups (Specific Action vs. Pointing). The model revealed an effect of Condition for both learning groups individually.

The model included the sum-coded fixed factors Group (Specific Action vs. Pointing), Performance (High vs. Low), Condition (Match vs. Mismatch), and ROI, as well as their interaction, with random intercepts for Participant and Item. The model revealed an interaction of Condition:Performance group (*β* = 4.12, *SE* = 4.63, *t* = 8.888, *p* < 0.001). The data were modeled independently thereafter for each Performance group.

##### High performance learners.

We performed analyses on High performance participants across the two learning groups (Specific Action vs. Pointing), using the same subset of frontocentral electrodes listed in the EEG Data Analysis section. The model included the sum-coded fixed factors Group (Specific Action vs. Pointing), Condition (Match vs. Mismatch), ROI (Midline, Left, Right), as well as their interactions, with random intercepts for Participant and Item. The model revealed an interaction of Condition:Group (*β* = 6.29, *SE* = 4.72, *t* = 13.310, *p* < 0.001). The data were modeled independently thereafter for each experimental group (Specific Action vs. Pointing), using the same model structure as above without the fixed factor Group.

**Figure 5.**
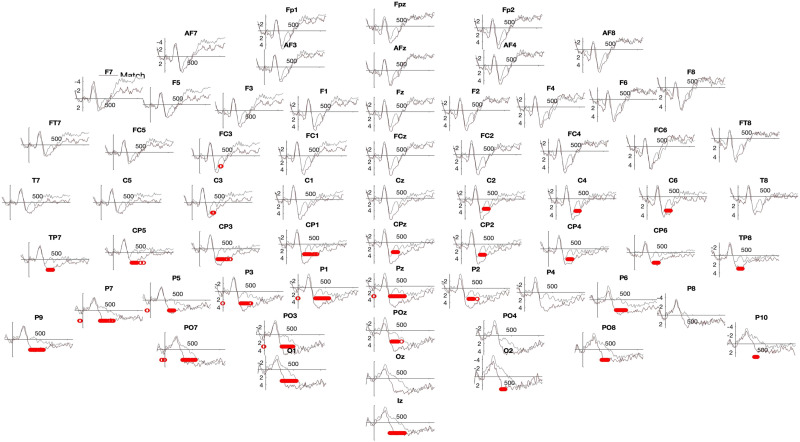
Post-training Pointing group—High performance learners. Significant two-tailed permutation tests are indicated in red.

**Figure 6.**
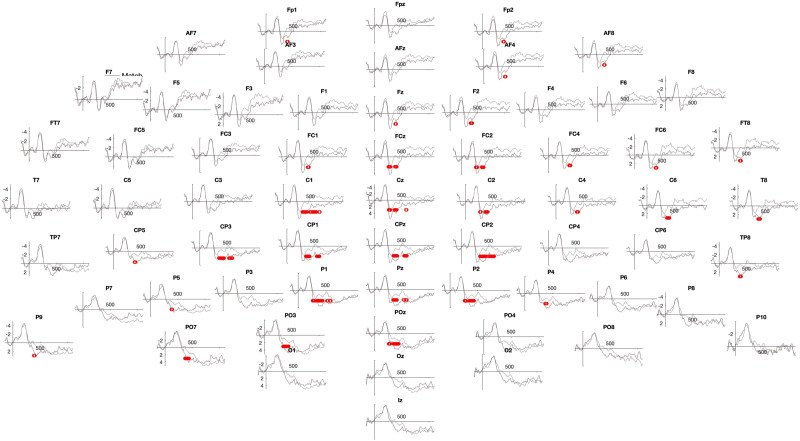
Post-training, Specific Action group—High performance learners. Significant two-tailed permutation tests are indicated in red.

- **Pointing group**: We ran the same model as above, without the factor Group. The model revealed an effect of Condition (*β* = 8.10, *SE* = 6.52, *t* = 12.432, *p* < 0.001) that did not interact with ROI (*β* = 3.18, *SE* = 8.20, *t* = 0.388, ns & *β* = 1.65, *SE* = 1.09, *t* = 0.152, ns). See Figure 5.
- **Specific Action group**: The same model revealed a significant effect of Condition (*β* = 4.27, *SE* = 6.80, *t* = 6.284, *p* < 0.001) that did not interact with ROI (*β* = −9.62, *SE* = 8.49, *t* = −0.113, ns & *β* = 1.32, *SE* = 1.13, *t* = 0.117, ns). See Figure 6.

##### Low performance learners.

**Figure 7.**
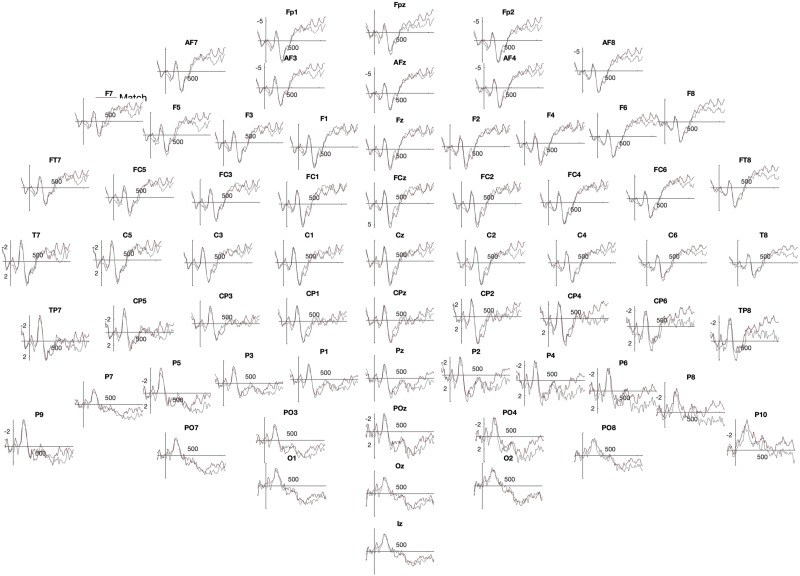
Post-training, both groups—Low performance learners.

We performed analyses on Low performance participants across the two learning groups (Specific Action vs. Pointing), using the same subset of frontocentral electrodes listed in the EEG Data Analysis section. The model included the sum-coded fixed factors Group (Specific Action vs. Pointing), Condition (Match vs. Mismatch), ROI (Midline, Left, Right), and their interactions, with random intercepts for Participant and Item. No effect of Condition was found (*β* = −1.18, *SE* = 6.16, *t* = 0.851, ns). See Figure 7.

#### Passive listening task: Time frequency *t* tests

In order to further test the hypothesis of greater motor activation for test compared to filler verbs, post-training, we performed paired *t* tests comparing pre- versus post-training activity in the control group for filler verbs, in the control group for learned verbs, in the test group for filler verbs, and in the test group for learned verbs. Once again, no significant activity emerged in either the mu, high beta, or low beta frequency bands according to our statistical analysis. Time frequency figures illustrating these analyses are included in the Supporting Information, available at https://doi.org/10.1162/nol_a_00132.

## DISCUSSION

The current study explored the neural correlates of embodied learning in a pre–post test word learning paradigm. Our primary aims were to investigate whether learning action verbs in an L2 is enhanced by action performance, and, if so, whether this enhancement is indexed by motor activation during word processing, post-training. Participants learned L2 words by either observing and performing specific actions that were compatible with the semantic content of words or simply observing these actions and pointing to objects, in an interactive virtual environment. We measured vocabulary acquisition using behavioral and EEG measures as participants performed a match–mismatch task pre- and post-training as well as a post-training production and translation task. Based on previous results showing that performing actions that are congruent with learned meaning facilitates word learning (Engelkamp & Krumnacker, 1980; Johnson-Glenberg & Megowan-Romanowicz, 2017), we hypothesized that our manipulation would lead to semantic integration as indexed by an N400 effect for mismatch versus match trials post-training in both groups. We also expected that if performing actions strengthens the association between newly learned verbs and motor actions more than simply observing actions and pointing, the N400 effect would be larger for the Specific Action group. Behavioral analysis for accuracy scores for the match–mismatch task revealed that, overall, both experimental groups learned the L2 vocabulary, but no differences emerged between the two groups. In relation to the neural response, analyses of the match–mismatch task at pre-training did not reveal any differences in the ERP trace between conditions, in line with predictions. Contrary to our expectations, no differences emerged post-training between these two conditions in the N400 time window for either group (Specific Action vs. Pointing) in our planned comparisons.

Participants also performed a passive listening task during which we measured motor activation via EEG as they listened to learned verbs and fillers pre- and post-training. In line with embodied theories, we expected our manipulation (training), which involved action performance versus observation and pointing, to mimic real-word embodied learning and link motor activity to the semantic meaning of the verbs. As viewing actions has been shown to activate the motor cortex in the same manner (albeit less) as action performance, we expected both learning conditions to lead to motor activation during passive listening post-training and for this activation to be greater post-training for verbs learned with specific actions. Pre-training, no motor resonance as indexed by mu, low beta, or high beta was seen for either group. Once again, contrary to our expectations, our results did not reveal greater motor activation for learned words compared to fillers for either experimental group (Specific Action vs. Pointing) post-training.

Embodied theories attribute a key role to motor processes in cognition and assume that semantic, and hence linguistic, representations are multimodal and grounded in real-world experience (Barsalou, 2008; Pulvermüller, 2005). Sensory and motor systems have been shown to be recruited during lexical processing, during both L1 and L2 action language processing (Hauk et al., 2004; Pulvermüller, 1999, 2005; Thompson-Schill, 2003; Zappa et al., 2019). According to the *correlational learning principle*, the co-occurrence of action perception and meaning results in the common firing of neurons, forming neural distributed neural networks that subserve semantic processing (Pulvermüller, 1999). Although a handful of previous EEG studies have found motor activation during action language processing in an L2, L2 speakers who show these effects were highly proficient in the L2 (Vukovic & Shtyrov, 2014). The only study to examine motor activation during the early phases of novel word learning, observed a pattern of activation that could be interpreted as motor activation immediately post-training (same day) but which became less interpretable the next day (Fargier et al., 2012). Indeed, motor activation during L2 processing seems to be contingent on proficiency and real-word experience with the L2. Seminal memory studies that show the enactment effect used the first language (Engelkamp & Krumnacker, 1980; Engelkamp & Zimmer, 1984). Hence, motor activation has been shown to be robust in the L1, likely due to innumerous instances of coupling of action performance and viewing over time. Our manipulation involved action observation with pointing versus action observation and performance of specific actions during two 30 min sessions, over two consecutive days. Our results indicate that this exposure did not result in a sufficiently robust motor trace, or motor-to-semantic association. Future studies would benefit from having participants learn novel words over a longer learning period, which would allow them to form better associations between actions and learned words.

Participants showed a great deal of variability in learning, as revealed by performance on the written production and translation tasks at the post-test. This was true for both the specific action and the control groups. We can note that vocabulary acquisition was rather difficult, as participants had to learn 12 verbs in a completely unfamiliar language (Serbian) that is unrelated to their native language (French) after only 10 exposures over the two learning sessions. While all of the Serbian words were phototactically legal in French, they bore no relationship to their French counterparts. Cognitive abilities (Kroll et al., 2002) have been shown to play an important role in L2 learning. For example, a handful of studies have found that working memory affects L2 processing in general (Reichle et al., 2016), and word learning in particular (Mota & Verçosa, 2007), including cross-modal working memory binding abilities (Wang et al., 2017). Although we did not test participants for such individual differences, it is possible that they could underlie learning success, which could in turn have affected electrophysiological results (N400 effect for match–mismatch task and motor activation during passive listening task).

The extreme variability in participants’ vocabulary learning could have directly impacted the neural pattern obtained for the group analyses. Otherwise stated, it is possible that participants who showed little success in learning, as revealed by their ability to produce and translate the vocabulary, could statistically eliminate predicted effects in those who learned the action verbs, in both experimental groups. To explore this, we separated participants into high performance and low performance learners, based on their ability to produce and translate the action verbs post-training. We performed statistical analyses on the match–mismatch task, which revealed an interaction between learning Performance and Condition. The results for the match–mismatch task post-training matched our initial expectations for both learning groups. For high performance learners, an N400 effect emerged for mismatch compared to match in both the Specific Action and the Pointing groups. No such effect was found for low performance learners in either experimental group.

These results show that the high performance learners succeeded in quickly learning an L2 vocabulary, as demonstrated by both the establishment of an N400 effect for newly learned words and by their ability to translate and produce the learned vocabulary. Several studies have shown N400 modulations related to learning an L2 and artificial languages following extended L2 training (Chun et al., 2012; McLaughlin et al., 2004; Soskey et al., 2016; Yum et al., 2014), but only a handful have shown these effects during early stages of encoding (Zappa et al., 2022). Although L1 studies have shown a very rapid emergence of the N400 for novel words presented in highly constraining contexts (Borovsky et al., 2012; Mestres-Missé et al., 2007), ours is the first to show this effect as a result of more naturalistic embodied L2 word learning using VR, after two 30 minute sessions of training.

As concerns embodied learning, it is an open debate whether physically performing congruent actions as opposed to simply viewing actions is advantageous for one-to-one mapping between labels and actions. Indeed, a handful of studies have shown that simply viewing actions that are congruent to semantic content provides a learning advantage compared to viewing pictures or receiving verbal input alone (Macedonia et al., 2019; Sweller et al., 2020). In fact, Sweller and colleagues compared viewing gestures while learning to perform them and found no differences between conditions. We expected that if action performance creates a stronger motor trace (i.e., the enactment effect or embodied learning), the Specific Action group, who both observed and performed congruent actions during learning, would achieve better semantic integration of the taught words compared to the Pointing group. On the other hand, if performance does not lead to better integration than observation, the Pointing group, who only observed actions and systematically pointed to virtual objects, should have integrated the taught words as well as the Specific Action group. Our results for the high performance learners do not reveal significant differences between the Pointing and Specific Action groups.

Overall, these results differ from both behavioral studies showing the enactment effect (Engelkamp & Krumnacker, 1980; Engelkamp & Zimmer, 1984; Moskowitz, 1976; Öttl et al., 2017; Quinn Allen, 1995) and those of several fMRI studies, outlined in the Introduction, showing that learning novel words is better supported when accompanied by self-performed congruent gestures compared to viewing images, or being exposed to only verbal content (Macedonia et al., 2019; Mayer et al. 2015). This improved performance is generally linked to the creation of an *embodied representation* based on greater activity in specialized visual and motor areas for words learned with picture visualization or self-performed actions (Mayer et al., 2015) or more distributed sensorimotor networks, for more learning modalities (Macedonia et al., 2019). Instead, our results suggest that action observation is as effective as action performance (Kormi-Nouri, 2000; Macedonia et al., 2019; Sweller et al., 2020).

As regards the passive listening task, the statistical power analyses we performed called for a sample size of 42 participants for the within-subjects comparisons in the time-frequency analyses. Given that we had 30 high performance participants in the Specific Action group and 25 in the Pointing group, we were unable to perform time-frequency analyses on these specific groups, due to lack of power. Nonetheless, the semantic effects we found point to a clear contribution of both action performance and observation to L2 word learning, in line with theories of embodied semantics, which claim that simulation is involved in language processing and learning.

### Caveats

As pointed out above, the great variability in learning success in both experimental groups is likely due to individual differences for which we did not control, such as executive and working memory. The main caveat of the current study is not taking these factors into account. Future studies would benefit from testing for individual differences pre-training and from having participants perform an initial non-language-related learning task to control for learning abilities. Another caveat is that both experimental conditions involved viewing actions, which induces motor activation. Including a control condition which does not involve any direct motor activation, such as rote learning using still images, would have allowed us to better link induced motor activation to learning success. It would also have allowed us to draw finer comparisons between induced motor activation during training and motor activation post-training during action verb processing. This comparison would tease apart motor effects that are a direct result of motor activation during L2 learning while observing and/or performing actions, and those that are already associated with L1 translations of learned verbs.

### Conclusion

Within the framework of embodied semantics, the current study examined whether action verbs learned with specific actions would lead better L2 verb learning and produce more motor activation post-training.

Declared analyses failed to show either semantic or motor effects post-training, most likely due to a high level of variability in learning success across experimental groups. Exploratory analyses revealed that learners in both groups who were able to translate and produce verbs behaviorally showed semantic effects post-training. These effects did not differ according to the learning condition. Semantic effects add electrophysiological evidence to rapid L2 word learning, supported by observing or performing actions, observed in numerous behavioral studies. These results lend support to embodied theories that claim that semantic meaning is represented multimodally. Furthermore, ours is the first study to use VR to induce motor activation during L2 learning and validates the experimental use of such a setup to explore other aspects of embodied L2 learning.

## ACKNOWLEDGMENTS

The work reported here was supported by grants from Institut Convergence (ILCB), Labex (BLRI), the Excellence Initiative of Aix-Marseille University (A*MIDEX), and the European Union’s H2021-MSCA-IF-2021 (project BraSILL No. 101062671).

## FUNDING INFORMATION

Cheryl Frenck-Mestre, BLRI, Award ID: ANR-11-LABX-0036. Cheryl Frenck-Mestre, ILCB, Award ID: ANR-16-CONV-0002.

## AUTHOR CONTRIBUTIONS

**Ana Zappa**: Conceptualization: Lead; Funding acquisition: Equal; Investigation: Supporting; Methodology: Lead; Software: Supporting; Writing – original draft: Lead. **Deidre Bolger**: Data curation: Supporting; Methodology: Supporting; Writing – review & editing: Supporting. **Jean-Marie Pergandi**: Conceptualization: Supporting; Formal analysis: Supporting. **Raphael Fargier**: Conceptualization: Supporting; Methodology: Supporting. **Daniel Mestre**: Conceptualization: Supporting; Methodology: Supporting; Supervision: Supporting. **Cheryl Frenck-Mestre**: Conceptualization: Supporting; Funding acquisition: Equal; Supervision: Supporting; Writing – original draft: Supporting.

## DATA AND CODE AVAILABILITY STATEMENT

Anonymized raw data and digital study materials are available on Zenodo: 10.5281/zenodo.10301899. The approved Stage 1 protocol can be found on the Open Science Framework: https://osf.io/futns/?view_only=951661c9eac7483bbfa80128fe67e5bb.

## Supplementary Material


