# Supplementary material for: The Neural Correlates of Embodied L2 Learning: Does Embodied L2 Verb Learning Affect Representation and Retention?
Source: Neurobiol Lang (Camb). 2024 Jun 3;5(2):360–84. doi: 10.1162/nol_a_00132 (PMC11192445; doi:10.1162/nol_a_00132)
Supplement: Supplementary file 1 [file nol-5-2-360-s001.pdf]

Supporting Information

Time frequency figures illustrating *t* test (4.2.3)

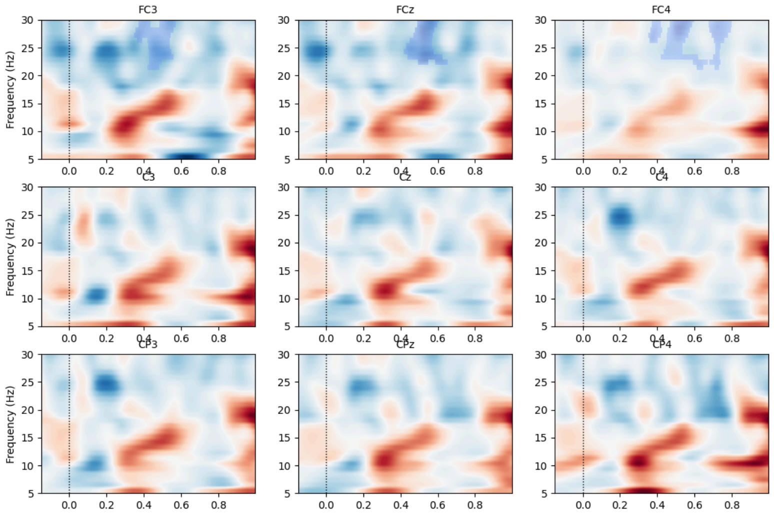

Fig.1 Paired t-test for Control group, Post minus Pre-training activity, for the Filler verbs

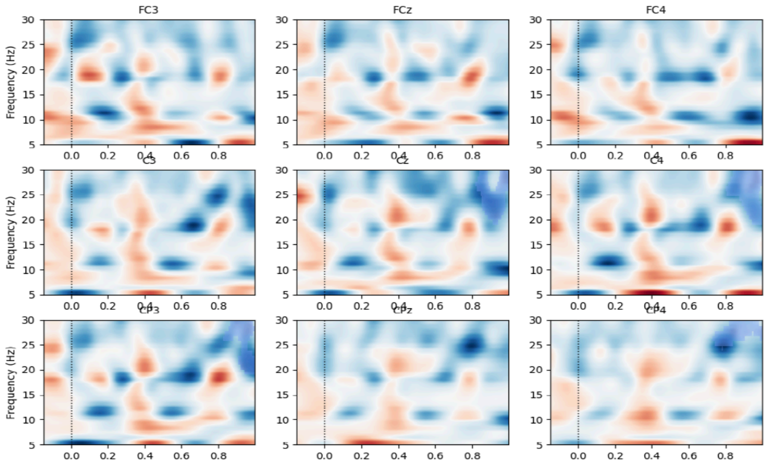

Fig.2 Paired t-test for Control group, Post minus Pre-training activity, for the Learned verbs

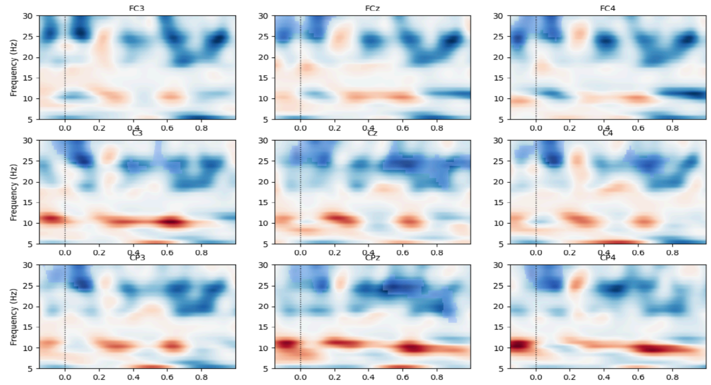

Fig.3 Paired t-test for Test group, Post minus Pre-training activity, for the Filler verbs

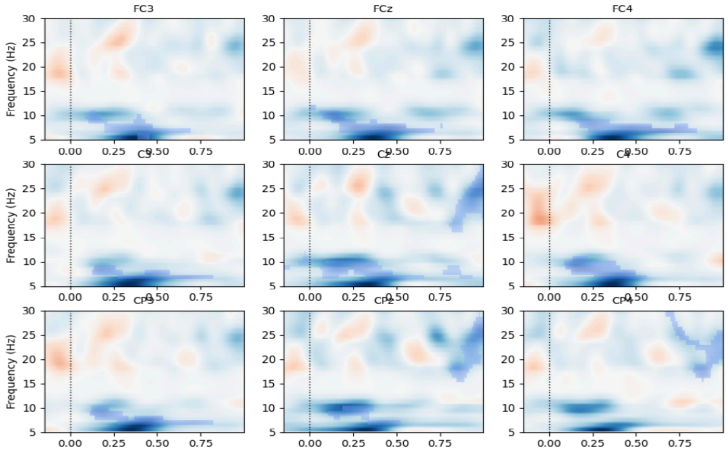

Fig.3 Paired t-test for Test group, Post minus Pre-training activity, for the Learned verbs
